# Early Nutrition during Hospitalization in Relation to Bone Health in Preterm Infants at Term Age and Six Months Corrected Age

**DOI:** 10.3390/nu13041192

**Published:** 2021-04-05

**Authors:** Alexandra K. Calor, Dana F.J. Yumani, Mirjam M. van Weissenbruch

**Affiliations:** Amsterdam UMC, Department of Pediatrics, VU University Medical Center, 1081 HV Amsterdam, The Netherlands; d.yumani@amsterdamumc.nl (D.F.J.Y.); m.vanweissenbruch@amsterdamumc.nl (M.M.v.W.)

**Keywords:** nutrition, preterm infants, bone health, bone mineral content, bone mineral density

## Abstract

Aim: to evaluate the potential association of macronutrient intake in the first postnatal weeks on bone mineral content (BMC) and bone mineral density (BMD) in extremely and very preterm infants. Methods: fifty-eight extremely and very preterm infants were included. Daily macronutrient intake was calculated in g kg^−1^ day^−1^ from birth up to 36 weeks postmenstrual age. A dual-energy X-ray absorptiometry whole body scan was used to assess BMC and BMD in preterm infants at term corrected age (TCA) and six months corrected age (CA). Results: fat intake (g kg^−1^ day^−1^) in the first four postnatal weeks was positively associated with BMC and BMD at TCA. At six months CA, protein and fat intake (g kg^−1^ day^−1^) in the first weeks of life were both individual predictors for BMD. Fat intake (g kg^−1^ day^−1^) in the first four postnatal weeks was significantly associated with BMC at six months CA. Conclusion: the association of macronutrient intake in the first postnatal weeks on BMC or BMD, at TCA and six months CA, suggest that early nutritional intervention immediately after birth and during early infancy is important for bone health in the first months of life.

## 1. Introduction

Both early postnatal life and the third trimester of the pregnancy are crucial factors for skeletal growth [1,2]. During the last trimester, the fetus has a higher rate of skeletal growth [1]. Consequently, preterm infants with a gestational age (GA) below 28 weeks have an increased incidence of impaired skeletal mineralization making them more susceptible to fractures [1,3,4]. The impaired mineralization of the skeleton in premature infants is a result of many nutritional and biochemical factors [1].

Previous research reports that children between the age of 5 and 9 years who were born prematurely have both lower spine mineral density and mineral content compared to term age infants [5,6]. Preterm infants have lower bone mineral content (BMC) and lower bone mineral density (BMD) at birth and at 40 weeks corrected age compared to term infants [7,8,9]. BMC and BMD increase substantially during the first 36 months after birth. Evaluation of BMC and BMD in preterm infants requires data on healthy growing infants and toddlers of the same age to provide a reference. Unfortunately, data on BMC and BMD in infants are limited [10].

In the pediatric population, dual-energy X-ray absorptiometry (DXA) has been described as the ideal technique to accurately assess the BMC due to the quick scan time and low radiation exposure [11]. While DXA is a frequent used technique in assessing BMD and BMC in children and adults, reference data on children below the age of 3 years are scarce [10]. One study reports a 5-fold increase in lumbar BMC and 2-fold increase in BMD between 24 and 36 months of age. They also point out that BMC was significantly greater in males than females during that same period of time and that race was of no influence on BMC or BMD [10].

Undernutrition and low intake of calcium, phosphorus, and vitamin D during early life has been linked to osteoporosis later in life [2,6,7,12]. While human milk is essential for preterm infants, it may contain insufficient bone-forming minerals for their needs and therefore makes them more at risk for bone fractures [7,13,14]. Nevertheless, the proportion of unsupplemented human milk in neonatal life is positively associated with bone mineral content and whole body bone size in later life [6].

A few animal studies have shown that a high fat diet, especially one with saturated fatty acids, results in a stimulation of bone resorption and affects the osteoblastogenesis [15,16]. Other studies have inconsistent findings on the association of fat intake and bone maturation [17,18,19]. The effects of a high carbohydrate diet in animal studies and protein intake in adult studies on bone health have shown conflicting results [16,20,21].

The aim of this study is to investigate the potential association of macronutrients in the first weeks of life, and BMC and BMD in extremely and very preterm infants at term age and six months corrected age, with the use of a whole body DXA scan. Furthermore, we will evaluate the difference in macronutrient intake in extremely and very preterm infants, by reason of the longer time ex utero of the extremely preterm infants, with a longer exposure to the nutritional intervention of clinicians.

## 2. Methods

### 2.1. Study Population

The study cohort consisted of 58 preterm infants born between 2015 and 2018 with a gestational age of 24 to 32 weeks, without substantial congenital anomalies, and admitted to the neonatal intensive care unit of the Amsterdam University Medical Center (Amsterdam UMC), location VU University Medical Center. These preterm infants were part of the “Nutrition in relation to the endocrine regulation of preterm growth” (NUTRIE) study. The NUTRIE study is a longitudinal observational study that describes the role of nutrition in relation to the regulation of endocrine hormones and growth. Written informed consent was obtained within the first postnatal week. The Medical Ethics Committee of the VU University Medical Center approved this study and is listed at the Dutch Trial register (www.trialregister.nl, NTR5311, 21-07-2015).

### 2.2. Study Procedures

Within 24 h after birth, all preterm infants were admitted to the Neonatal Intensive Care Unit of the Amsterdam UMC, location VU University Medical Center. As soon as the infants were in good clinical condition with a postmenstrual age (PMA) of at least 30 weeks and a weight of at least 1000 g, they were transferred to step-down units in referral hospitals. Obstetric data, clinical condition, and nutrient intake were collected from birth up to 36 weeks PMA from hospital records. Weight was measured once a week until term corrected age (TCA) using an electronic scale to the nearest gram, standard deviation scores (SDS) of weight were calculated according to Fenton [22]. The corrected age of preterm infants is the age the preterm would have been if born at term (40 weeks). It is calculated as followed: actual/chronological age subtracted by the number of weeks of prematurity [23]. Weight SDS scores were based on the World Health Organization standard charts at TCA and six months corrected age (CA) [24]. At TCA and six months CA, all preterm infants received a DXA scan to assess BMD and BMC. Preterm infants that were only able to receive one DXA scan due to hospital admission or cancelling of the outpatient visit, were still included in this investigation.

### 2.3. Nutrition

After birth, total parenteral feeding was administered and minimal enteral feeding was started as described previously [25]. During total parenteral feeding, the infants had an energy intake of 85–100 kcal kg^−1^ day^−1^, a fat intake of 3–3.5 g kg^−1^ day^−1^, and a protein intake of 3–4 g kg^−1^ day^−1^. Full enteral feeding of 160 mL kg^−1^ day^−1^ with a total protein intake of 3.5–4.5 g kg^−1^ day^−1^ and a total energy intake of 110–140 kcal kg^−1^ day^−1^ was aimed to be achieved within 7–10 days after birth. The preterm infants received human milk for the most part. In case of unavailable “own mother’s milk” or insufficient mother’s milk, up to 32 weeks PMA, donor milk was administered followed by preterm starter formula, up until discharge. When donor milk was declined, starter preterm formula was administered. Additionally, after the first week of life, all preterm infants received 400–600 IU cholecalciferol (Vitamin D) per day.

Neonatologists were aiming to reach a weight SDS greater than −1SD gain with a weight gain of 15–20 g kg^−1^ day^−1^. Once the enteral intake reached 100 mL kg^−1^ day^−1^ Nutrilon Nenatal Breast Milk Fortifier was added. If poor growth was observed, the intake would be increased to a maximum of 180 mL kg^−1^ day^−1^ when tolerated by the preterm infant. In the case of persisting poor growth, 1% Nutrilon Nenatal Protein Fortifier was added to the fortified milk. If weight gain did not occur with fortification, up to 4% of Calogen Fat Emulsion was added.

Table 1 outlines the composition of enteral [13,26] and parenteral intake per 100 mL [27].

### 2.4. DXA

The dual-energy X-ray absorptiometry whole body scan (Hologic 4500-A Hologic Inc., Bedford, MA, USA) was utilized to assess BMC and BMD, in preterm infants at term CA and six months CA. The DXA device uses a tube that alternately produces two x-ray energies, 70 kilovolts (low), and 140 kilovolts (high) energy photon beams [28]. The average radiation produced by this machine has an effective dose equivalent around 5 microsieverts (μSv), which is considered a low exposure with a dose smaller than the daily background radiation exposure per day [28,29]. The pediatric software calculates the data of BMC in grams and BMD in g/cm^2^.

The scanning routine was performed by positioning the naked infant wrapped in a blanket on the scanning surface without the use of sedatives. The average scanning time was around 10 min [30] and was performed by a single experienced investigator.

### 2.5. Potential Confounders

Preterm infants struggle to achieve independent oral feeding, resulting in an inadequate nutritional status [31]. Comorbidities in preterm life have an even more complicated effect on the nutritional intake and growth. The following comorbidities were considered potential confounders in the possible association between early nutrition and bone health:

- Necrotizing enterocolitis: according to the modified Bell’s staging criteria; definite: necrotizing enterocolitis (NEC) from stage IIA onwards [32].
- Bronchopulmonary dysplasia: lung injury from oxygen therapy and mechanical ventilation; 28 days of oxygen administration [33].
- Late onset sepsis: neonatal sepsis occurring after 3 days of age [34].

Moreover, the gender and ethnicity of the infant, birthweight, weight at TCA and six months CA, as well as the gestational age were considered potential confounders.

Previous research has shown that males have greater BMC and BMD during infancy than females [35]. Additionally, black infants have greater BMD than white infants [35]. Another study with prepubertal children with an age range of 6–11 years supports the difference in race and gender on bone health [36]. Preterm infants with a birthweight less than 1500 g have lower lumbar spine BMC and BMD compared with term infants at the age of 7 years [37]. Furthermore, a recent study in 5-year-old children noted that an increased body size is associated with higher concurrent BMD [38]. These results suggest an association of both weight and concurrent weight on bone health.

### 2.6. Statistical Analysis

Ordinal data of extremely preterm infants and very preterm infants were compared using a Mann–Whitney U Test and nominal data were compared using a Chi-Square (X^2^) test. No assumptions were violated when analyzing the groups using the Mann–Whitney U Test; ordinal data in two independent groups with independence of observations. Paired t-tests were used to compare BMD and BMC of extremely and very preterm infants over time. The dependent variables were continuous and normally distributed. Stepwise multiple regressions were carried out to evaluate the influence of macronutrient intake, gestational age, gender, ethnicity, birthweight, weight at TCA, weight at six months CA, necrotizing enterocolitis, bronchopulmonary dysplasia, and late onset sepsis on BMC and BMD. All variables were included in the analysis. However, only the significant models with corresponding variables were included in the results (Tables 5 and 6). The assumptions were met because of the linear between the dependent and independent variable, with the same variance of the residuals, homoscedasticity, and a normally distributed model. A *p*-value of <0.05 was considered statistically significant. All statistical analyses were conducted using the IBM^®^ SPSS^®^ Statistics for Windows, Version 26.0 (IBM Corp., Armonk, NY, USA).

## 3. Results

Baseline characteristics of the preterm infants are outlined in Table 2. Extremely preterm infants had a mean GA of 26.9 weeks (24–27 weeks) and the very preterm infants a mean GA of 29.8 weeks (28–31 weeks). There was no significant difference in ethnicity between these two groups. Birthweight in grams (*p* < 0.001) and SDS (*p* = 0.049) and weight in SDS at TCA (*p* = 0.029) were significantly different between the groups. Of the different comorbidities, bronchopulmonary dysplasia was more common in extremely preterm infants than very preterm infants, *p* < 0.001 (Table 2).

### 3.1. Nutrient Intake in the First Weeks of Life

The mean (SD) length of parenteral feeding in days in extremely preterms and very preterms was respectively 12.9 (8.7) and 16.3 (9.6). In the second week of life, there was a 1.6-fold increase in energy intake in kcal kg^−1^ day^−1^ in extremely and very preterm infants compared to the first week of life. The protein intake was 1.5 times higher in the second week of life in extremely preterms, while very preterm infants had a 1.7-fold increase in protein intake (*p* = 0.606). The carbohydrate intake in kcal kg^−1^ day^−1^ was significantly higher in extremely preterm infants compared with very preterm infants in the second postnatal week (*p* = 0.017).

**Figure 1:**
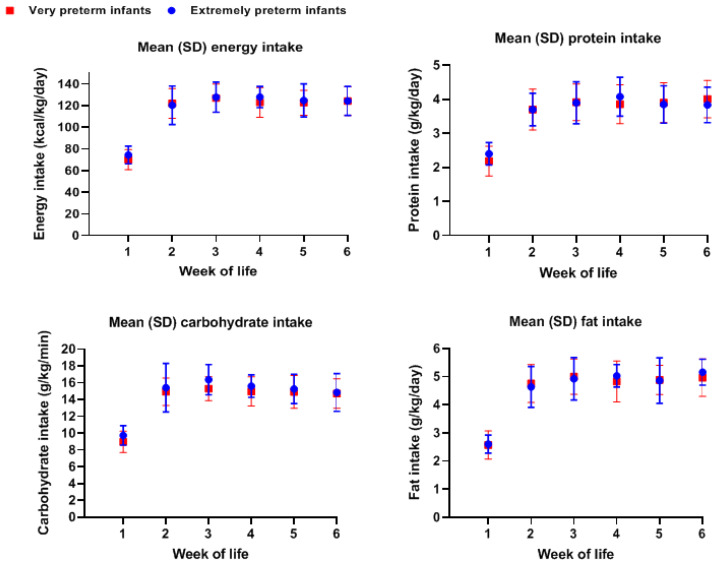
Nutrient intake in extremely and very preterm infants during the first weeks of life.

There was a steady intake of all macronutrient in both groups after the second until the sixth postnatal week (Figure 1). There was no significant difference in macronutrient intake in percentages of energy between extremely and very preterm infants during the first four postnatal weeks (Table 3). The macronutrient distribution was similar during these first weeks, with carbohydrates being the larger part of the percentage of energy intake (Table 3).

### 3.2. BMD and BMC in Extremely and Very Preterm Infants

At TCA, 14 extremely preterm infants and 23 very preterm infants had a DXA scan. Of these preterms, seven extremely preterm infants and nine very preterm infants also had a scan at six months CA. In addition, a DXA scan was performed in another 21 preterm infants; seven extremely preterm and 14 very preterm infants at 6 months CA. Therefore, a total of 16 preterm infants had two DXA scans and 42 preterm infants had one scan.

There is no significant difference between extremely and very preterm infants in BMC (g) and BMD (g/cm^2^) at TCA (Table 4). At six months CA, BMD, and BMC were equally insignificant between the groups. Extremely preterm infants had an increase of 67.5 g of BMC from TCA to 6 months CA (*p* = 0.29), while very preterm infants had an increased BMC of 59.2 g during this time (*p* = 0.84, Table 4). The increase of BMD from TCA until six months CA was not significant in both groups (Table 4).

### 3.3. Macronutrient Intake in the First Postnatal Weeks and BMC at TCA

Energy intake (kcal kg^−1^ day^−1^) in the first four weeks of life was positively associated with BMC at TCA (Table 5, model 1). GA, gender, ethnicity, and comorbidities did not add significantly to the prediction of BMC. Into more detail, of the macronutrients, only fat intake (g kg^−1^ day^−1^) in the first four postnatal weeks was positively associated with BMC at TCA (Table 5, model 2). Late onset sepsis (LOS) was a significant contributor to the association (*p* = 0.04).

### 3.4. Macronutrient Intake in the First Postnatal Weeks and BMD at TCA

Energy intake (kcal kg^−1^ day^−1^) and fat intake (g kg^−1^ day^−1^) in the first four postnatal weeks was positively associated with BMD at TCA (Table 5, model 3 + 4). Carbohydrate and protein intake (g kg^−1^ day^−1^) in the first four weeks of life were not associated with BMD at TCA.

### 3.5. Macronutrient Intake in the First Postnatal Weeks and BMC at Six Months CA

Energy intake in kcal kg^−1^ day^−1^ in the first four weeks of life and weight SDS at six months CA are associated with BMC at six months CA (Table 6, model 1). Protein intake (g kg^−1^ day^−1^) in the first four postnatal weeks and weight SDS at six months CA were significantly associated with BMC at six months CA (Table 6, model 2). Fat intake in g kg^−1^ day^−1^ was close to, but not quite statistically significant, to BMC at six months CA (Table 6, model 3).

### 3.6. Macronutrient Intake in the First Postnatal Weeks and BMD at Six Months CA

Both protein and fat intake (g kg^−1^ day^−1^) in the first four postnatal weeks were positively associated with BMD at six months CA (Table 6, model 5 + 6). Additionally, the energy intake (kcal kg^−1^ day^−1^) in the first four weeks of life was associated with BMD at six months CA (Table 6, model 4). Gender, birthweight SDS, concurrent weight SDS and comorbidities were no significant variables in the association with BMD at six months CA.

## 4. Discussion

This study shows that there is no significant difference between extremely and very preterm infants in BMC (g) and BMD (g/cm^2^) at TCA and six months CA. In addition, there was a non-significant increase of BMC and BMD from TCA to six months CA in both groups. Energy intake in kcal kg^−1^ day^−1^ in the first four weeks of life was positively associated with BMC and BMD at TCA as well as six months CA. Mean fat intake (g kg^−1^ day^−1^) in the first postnatal weeks and LOS were significant predictors for BMC at TCA. Furthermore, fat intake in g kg^−1^ day^−1^ was associated with BMD at TCA. At six months CA, protein and fat intake (g kg^−1^ day^−1^) were positively associated with BMD and fat intake (g kg^−1^ day^−1^) with BMC. Gender and ethnicity were not associated with BMC and BMD at TCA or six months CA.

### 4.1. BMC and BMD in Preterm Infants

Extremely and very preterm infants in this study showed a 2-fold insignificant increase in BMC and 1.3-fold increase in BMD from TCA to six months CA. The study of Kalkwarf et al. [10] describe a greater increase in BMC in BMD in the first postnatal months. Our small sample size could be an explanation for the non-significant increase. Moreover, their study population consisted of term infants and a lumbar spine DXA scan was performed, while this study used a whole body DXA scan. Our results showed no association between gender and BMD or BMC, while Kalkwarf et al. report higher BMC in males. Literature on BMC and BMD in the first postnatal months in preterm infants is scarce, which makes comparing our results with only term infants difficult to interpret. However, van de Lagemaat et al. [39] describe a preterm population with a GA of 32 weeks or less and/or with a birthweight of 1500 g and fed with either post-discharge formula, term formula or human milk until six months CA. The results of this study also showed an increase in BMC in preterm infants fed with term formula or human milk at six months CA compared to TCA [39]. It is generally assumed that gain in BMC in early infancy has an impact on future bone health. Low BMC in infancy may persist into adulthood resulting in lower adult bone mass [40,41,42]. This may lead to a higher risk of osteoporosis in later life [43]. Therefore, it is plausible that increased gain in BMC in preterm infants as a result of early nutritional intervention with the desired composition and vitamin D immediately after birth and during early infancy, is important for adult peak bone mass. Interestingly, BMC and BMD values in our preterm infants at six months CA age (Table 4) were comparable with BMC and BMD values of term infants at birth (BMC 66.2 g, BMD 0.22 g/cm^2^) according to the findings of the systematic review and meta-analysis of Ramot et al. [4].

In the present study, BMC and BMD between extremely and very preterm infants was comparable at TCA and six months CA. Despite the fact that the extremely preterm infants had an average of 3 weeks less in utero for bone mineral accumulation, a difference was not apparent at TCA. This could be explained by the longer period of ex utero feeding, where fortifiers were added when poor growth was assessed. These fortifiers are relatively high in protein and fat and, therefore, result in higher energy intake. Additionally, at TCA extremely preterm infants received vitamin D supplementation for a longer period of time compared to very preterm infants. Previous data [44] describe both short- and long-term reduction of bone mineralization in infants with vitamin D deficiency.

A longitudinal study by Zhao et al. [45] on the trajectory of BMD described an independent association between low birth weight and BMD in the first 12 months of life in preterm infants. In our study, birthweight SDS was not a predictor for BMD. This might be explained by the larger sample size in their study, a study population containing solely Chinese infants and a birthweight measurement exclusively in kilograms.

### 4.2. Nutrient Intake in the First Postnatal Weeks on BMC and BMD in Preterm Infants Weeks

Based on our information, no previous studies describe the potential relation of macronutrients in the first postnatal weeks of preterm infants on BMC and BMD. Studies on nutrient intake and bone health mainly focus on the calcium, phosphorus, and vitamin D intake [6,7,12].

This study reveals a positive association of fat intake (g kg^−1^ day^−1^) in the first four weeks of life on both BMC and BMD at TCA. The relation of fat intake on bone health has been reviewed in mice studies [16]. These mice studies have shown conflicting results on the effect of fat intake on bone health, describing both beneficial and harmful effects of high fat diets [15,16,17,19]. Malvi et al. [18], however, have demonstrated that a high fat (24% crude fat) diet in young rats promotes peak bone mass after six months on this diet. The positive association of the fat intake on BMC and BMD could be explained by the high amount of medium chain fatty acids in preterm breast milk [46] and parenteral feeding [27]. Preterm infants are faced with an immature intestine that has less capacity to absorb long chain fatty acids [47]. This advantageous offer of medium chain fatty acids could have a positive effect on perinatal bone maturation. Nevertheless, these results should be interpreted carefully with this small sample size.

In the present study, both protein and fat intake (g kg^−1^ day^−1^) during the first postnatal weeks were related to BMD at six months CA. Studies on protein intake and bone health have also been conducted in healthy adults. A systematic review and meta-analysis by Darling et al. in 2009 reported a positive relation between protein intake and BMC and BMD [21]. However, the recently updated systematic review and meta-analysis by Darling et al. in 2019 [20] reported no association of dietary protein intake and bone health in adults. Previous research has shown that greater enteral intakes of energy and protein post discharge reduces the extent of early weight loss [48]. Infants born preterm with low birthweight (<1.5 kg) have lower BMC and BMD at age 7 compared to infants born term [5]. When poor growth was assessed, immediate nutritional intervention took place, resulting in accelerating growth of the preterm infants and could likely explain the positive relation with BMD at six months CA.

### 4.3. Sepsis in Early Life and Bone Health

The findings here indicates that LOS is a potential negative predictor for BMC (g) at TCA. A possible explanation for this association can be assigned to the change in nutrient demands in a state of illness. During illness, preterm infants have decrease absorption of nutrients and difficulties utilizing these nutrients [49]. Sick neonates are therefore more at risk of growth failure [49], thus potentially effecting bone health. Similar results have been reported in adult studies on illnesses and bone health, an association between sepsis and loss in BMD is observed [50,51].

### 4.4. Strengths and Limitations

To our knowledge, this is the first study to describe the relation of macronutrients in the first postnatal weeks on BMC and BMD at TCA and six months CA in preterm infants. These preterm infants were exclusively fed human milk during and after tapering off parenteral feeding. Our results do show a positive association between fat intake during the first postnatal weeks with BMC and BMD at TCA. At six months CA, fat and protein intake were associated with BMD and fat intake with BMC. Of all potential confounders, only LOS appeared to be a significant predictor for BMC (g) at TCA.

However, the findings of this study should be approached with caution, because of the observational nature of this study in a small sample size.

## 5. Conclusions

In conclusion, the associations between macronutrient intake in the first postnatal weeks on BMC or BMD, at either TCA or six months CA, in preterm infants do suggest that early nutritional intervention directly after birth and during early infancy is important for bone health in the first months of life. Longitudinal follow up of this cohort is needed, to investigate its impact on bone health later in life.

## Figures and Tables

**Table 1:** Composition of human milk and parenteral feeding per 100 mL.

Variables	OMM	OMM + BMF (4.4 g/100 mL)	DHM	DHM + BMF	PF
Energy (kcal)	68.5	83.8	60	75	66
Protein (g)	1.5	2.6	0.8	1.9	2.6
Carbohydrates (g)	7.3	10.0	7.5	10.2	8.9
Fat (g)	3.3	3.3	2.9	2.9	2

BMF: breast milk fortifier, DHM: donor human milk, OMM: own mother’s milk, PF: Parenteral Feeding.

**Table 2:** Characteristics of the preterm cohort.

	Extremely Preterm, *n* = 21	Very Preterm, *n* = 37	*p*-Value
	(<28 Weeks GA)	(28–<32 Weeks GA)	
Gestational age (weeks), mean (SD)	26.9 (0.9)	29.8 (1)	<0.001 ^1^
Gender, *n* (%)			
*Male*	10 (48)	20 (54)	0.119 ^2^
*Female*	11 (52)	17 (46)	
Ethnicity, *n* (%)			
*White*	14 (67)	29 (78)	0.774 ^2^
*Other*	7 (33)	8 (22)	
Birthweight (g), mean (SD)	963.9 (143.2)	1269.9 (306)	<0.001 ^1^
Birthweight SDS, mean (SD)	0.3 (0.6)	−0.1 (0.8)	0.049 ^1^
Weight at TCA (g), mean (SD)	4208.6 (669.1)	4065.2 (644.8)	0.856 ^1^
Weight SDS at TCA, mean (SD)	0.4 (1.1)	−0.1 (1.2)	0.029 ^1^
Weight at 6 months CA (g), mean (SD)	7099.2 (791.9)	7379.5 (864.1)	0.423 ^1^
Weight SDS at 6 month CA, mean (SD)	−0.6 (1.3)	−0.7 (1)	0.829 ^1^
Comorbidities, *n* (%)			
*BPD*	14 (67)	10 (27)	0.003 ^2^
*LOS*	11 (52)	13 (35)	0.157 ^2^
*NEC*	2 (1)	3 (1)	0.397 ^2^

^1^ Mann–Whitney U Test or ^2^ Chi-Square (X^2^) were used to compare groups. GA: gestational age, SDS: standard deviation scores, BPD: bronchopulmonary dysplasia, CA: corrected age, LOS: late onset sepsis, NEC: necrotizing enterocolitis, TCA: term corrected age.

**Table 3:** Macronutrient intake (% energy) in extremely and very preterm infants during the first four weeks of life.

		Extremely Preterm (*n* = 21)	Very Preterm (*n* = 37)
	Macronutrient Intake in % of Energy, Mean (SD)		
Week 1	Fat	17.6 (0.09)	18.4 (0.2)
	Protein	16.5 (0.09)	15.7 (0.2.)
	Carbohydrate	64.9 (0.16)	63.9 (0.6)
Week 2	Fat	19.2 (0.06)	19.1 (0.2)
	Protein	15.7 (0.14)	15.2 (0.1)
	Carbohydrate	63.4 (0.6)	62.2 (0.5)
Week 3	Fat	19.4 (0.05)	19.3 (0.2)
	Protein	15.6 (0.1)	15.6 (0.1)
	Carbohydrate	63.1 (0.6)	61.3 (0.5)
Week 4	Fat	19.6 (0.07)	19.2 (0.2)
	Protein	15.7 (0.1)	15.6 (0.09)
	Carbohydrate	61.9 (0.6)	62 (0.6)

**Table 4:** BMC and BMD in extremely and very preterm infants at TCA and 6 months CA.

		Extremely Preterm, *n* = 14	Very Preterm, *n* = 23	*p* Value ^1^
BMC (g), mean (SD)	TCA	62.2 (14.9)	56.3 (14.0)	0.69
	6 months CA	129.7 (35.1)	115.5 (24.9)	0.09
*p*-value ^2^		0.29	0.84	
BMD (g/cm^2^), mean (SD)	TCA	0.17 (0.02)	0.16 (0.02)	0.91
	6 months CA	0.23 (0.04)	0.21 (0.04)	0.88
*p*-value ^3^		0.29	0.84	

^1^ Comparison between groups (Mann–Whitney U test); ^2^ Change in BMC from TCA until 6 months CA (Paired t-test); ^3^ Change in BMD from TCA until 6 months CA (Paired *t*-test); BMC: Bone mineral content, BMD: bone mineral density, CA: Corrected age, TCA: term corrected age.

**Table 5:** Regression analyses of mean intake in the first four postnatal weeks as a predictor for BMC and BMD at TCA.

Variables	Model R^2^	Model *p*-Value	B (SE)	β	*p*-Value
**BMC (g) at TCA, *n* = 58**					
*Model 1*	0.496	0.004			
Constant			19.08 (47.89)		0.694
GA			−1.35 (1.28)	−0.172	0.303
Gender			−5.80 (4.33)	−0.210	0.193
Ethnicity			−0.97 (1.03)	−0.148	0.355
LOS			−7.79 (4.67)	−0.293	0.108
Mean energy intake in kcal kg^−1^ day^−1^ week 1–4			0.83 (0.25)	0.546	**0.002**
*Model 2*	0.46	0.001			
Constant			42.09 (40.82)		0.312
GA			−1.45 (1.25)	−0.185	0.259
LOS			−9.66 (4.47)	−0.363	**0.04**
Mean fat intake in g kg^−1^ day^−1^ week 1–4			14.40 (4.39)	0.508	**0.003**
**BMD (g/cm^2^) at TCA, *n* = 58**					
*Model 3*	0.45	0.021			
Constant			0.56 (0.05)		0.274
Gender			−0.01 (0.01)	−0.281	0.137
Ethnicity			−0.00 (0.00)	−0.154	0.372
BPD			0.01 (0.01)	0.25	0.186
NEC			−0.01 (0.01)	−0.221	0.368
Birthweight SDS			−0.00 (0.00)	−0.153	0.409
Mean energy intake in kcal kg^−1^ day^−1^ week 1–4			0.00 (0.00)	0.57	**0.019**
*Model 4*	0.474	0.014			
Constant			0.14 (0.06)		**0.022**
GA			−0.00 (0.00)	−0.226	0.217
Gender			−0.01 (0.01)	−0.271	0.14
Ethnicity			−0.00 (0.00)	−0.147	0.395
LOS			−0.01 (0.01)	−0.221	0.237
Birthweight SDS			−0.01 (0.00)	−0.192	0.302
Mean fat intake in g kg^−1^ day^−1^ week 1–4			0.03 (0.01)	0.687	**0.001**

BMC: Bone mineral content, BMD: bone mineral density, BPD: Bronchopulmonary dysplasia, GA: Gestational age, LOS: Late onset sepsis, NEC: necrotizing enterocolitis, SDS: Standard deviation scores, TCA: term corrected age.

**Table 6:** Regression analyses of mean intake in the first four postnatal weeks as a predictor for BMC and BMD at six months CA.

Variables	Model R^2^	Model *p*-Value	B (SE)	β	*p*-Value
**BMC (g) at 6 months CA, *n* = 58**					
*Model 1*	0.394	0.021			
Constant			99.07 (103.12)		0.345
GA			−4.08 (2.77)	−0.222	0.151
Ethnicity			1.12 (2.23)	0.08	0.621
Birthweight SDS			2.65 (6.40)	0.07	0.682
Weight SDS 6 m CA			10.62 (4.06)	0.417	**0.014**
NEC			19.37 (14.78)	0.232	0.201
Mean energy intake in kcal kg^−1^ day^−1^ week 1–4			1.28 (0.57)	0.406	**0.033**
*Model 2*	0.337	0.013			
Constant			127.43 (101.60)		0.219
GA			−3.69 (2.77)	−0.201	0.194
Weight SDS 6 m CA			11.33 (3.86)	0.437	**0.007**
NEC			13.56 (14.26)	0.163	0.349
Mean protein intake in g kg^−1^ day^−1^ week 1–4			30.59 (24.71)	0.354	**0.046**
*Model 3*	0.247	0.011			
Constant			161.67 (93.78)		0.095
GA			−4.47(2.76)	−0.243	0.115
Weight SDS 6 m CA			10.78 (3.87)	0.423	**0.009**
NEC			14.15 (14.66)	0.17	0.342
Mean fat intake in g kg^−1^ day^−1^ week 1–4			22.14 (11.22)	0.343	0.05
**BMD (g/cm^2^) at 6 months CA, *n* = 58**					
*Model 4*	0.238	0.036			
Constant			−0.05 (0.09)		0.609
Gender			0.015 (0.01)	0.199	0.218
NEC			0.03 (0.02)	0.276	0.143
Mean energy intake in kcal kg^−1^ day^−1^ week 1–4			0.01 (0.00)	0.501	**0.01**
*Model 5*	0.207	0.127			
Constant			0.02 (0.08)		0.767
Gender			0.02 (0.01)	0.298	0.109
NEC			0.03 (0.02)	0.232	0.224
Weight SDS 6 m CA			0.01 (0.01)	0.213	0.252
Mean protein intake in g kg^−1^ day^−1^ week 1–4			0.05 (0.02)	0.404	**0.036**
*Model 6*	0.177	0.105			
Constant			0.04 (0.07)		0.601
Gender			0.02 (0.01)	0.204	0.223
NEC			0.03 (0.02)	0.23	0.236
Mean fat intake in g kg^−1^ day^−1^ week 1–4			0.04 (0.02)	0.411	**0.037**

BMC: Bone mineral content, BMD: bone mineral density, BPD: Bronchopulmonary dysplasia, CA: Corrected age, GA: Gestational age, LOS: Late onset sepsis, NEC: necrotizing enterocolitis, SDS: Standard deviation scores, 6 m: six months.

## Data Availability

Due to the nature of this research, participants of this study did not agree for their data to be shared publicly, so supporting data is not available.

## Abbreviations

BMC	Bone Mineral Content
BMD	Bone Mineral Density
BPD	Bronchopulmonary Dysplasia
CA	Corrected Age
DXA	Dual-Energy X-Ray Absorptiometry
GA	Gestational Age
LOS	Late Onset Sepsis
NEC	Necrotizing Enterocolitis
PMA	Postmenstrual Age
SDS	Standard Deviation Scores
TCA	Term Corrected Age
